# Supplementary material for: The Effects of Endogenous Hormones on the Flowering and Fruiting of Glycyrrhiza uralensis
Source: Plants (Basel). 2019 Nov 17;8(11):519. doi: 10.3390/plants8110519 (PMC6918285; doi:10.3390/plants8110519)
Supplement: Supplementary file 1 [file plants-08-00519-s001.pdf]

plants

MDPI

Supporting Information

Effects of Endogenous Hormone on the Flowering and Fruiting of Glycyrrhiza uralensis

Binbin Yan [1,2], Junling Hou [3], Jie Cui [2], Chao He [2], Wenbin Li [3], Xiaoyu Chen [2], Min Li [1,*] and Wenquan Wang [2,*]

[1] Chengdu University of Traditional Chinese Medicine, Chengdu 610075, China; yb51598@126.com
[2] Institute of Medicinal Plant Development, Chinese Academy of Medical Sciences & Peking Union Medical College, Beijing 100193, China; jcui@implad.ac.cn (J.C.); hc891215@126.com (C.H.); cxy1033@163.com (X.C.)
[3] School of Chinese Pharmacy, Beijing University of Chinese Medicine, Beijing 100102, China; 602036@bucm.edu.cn (J.H.); wbli92@126.com (W.L.)
* Correspondence: limin@cdutcm.edu.cn (M.L.); wqwang@implad.ac.cn (W.W.); Tel.: +86-10-5783-3145 (M.L. & W.W.)

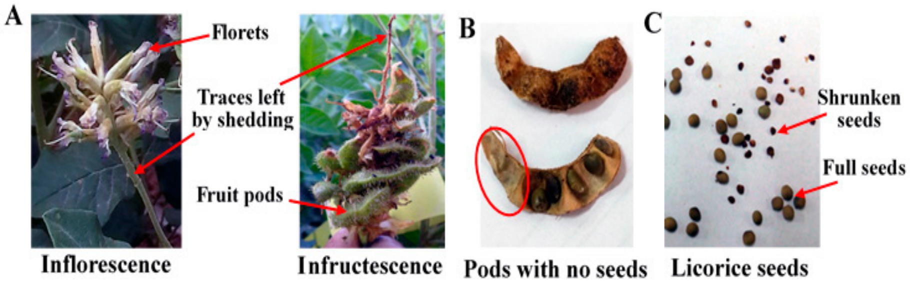

Figure 1. Flower and fruit falling, empty seed plants and licorice seeds. (A) Florets, fruit pods and traces after shedding; (B) Pods with no seeds; (C) Shrunken seeds and full seeds.

Table 1. Flowering and fruiting situations of plants in different sampling plots.

Age	Flowering and yield fruit / plants	No flowering / plants	Inflorescence fall off / plants
5y	52	28	20
5y	64	27	8
5y	70	19	11
7y	67	14	19
7y	70	27	4
7y	65	29	5
12y	73	21	6
12y	60	36	4
12y	69	25	6

Table 2. Rate of flower and fruit falling in different sampling plots（n=100）**.**

Age	5y	5y	5y	7y	7y	7y	12y	12y	12y
Rate of flower and fruit falling /%	55.63 + 9.00	51.96 + 8.00	56.78 + 9.00	44.43 + 6.00	47.38 + 5.00	44.02 + 5.00	48.97 + 8.00	51.48 + 6.00	48.46 + 6.00

Notes: Numbers denote the average rate of flower and fruit falling of 100 plants in each sample±SD.

$$\text{Rate of flower and fruit falling}(\%) = \frac{\text{Falling trace number of single infructescence}}{\text{Falling trace number of single infructescence} + \text{Fruit pods number of single infructescence}} \times 100\%$$

The number of traces refers to the number of traces left on the rachis after falling of flowers or pods.

Table 3. Rate of empty seeds, rate of shrunken seeds and TKW in different sampling plots (n=100)**.**

Age	5y	5y	5y	7y	7y	7y	12y	12y	12y
Rate of empty seeds /%	41.21 + 13.00	30.07 + 12.00	21.50 + 6.00	16.33 + 7.00	12.53 + 6.00	10.03 + 8.00	21.09 + 12.00	16.92 + 9.00	24.12 + 13.00
Rate of shrunken seeds /%	13.51 + 6.00	7.88 + 4.00	8.54 + 5.00	8.39 + 6.00	6.80 + 5.00	5.71 + 5.00	5.40 + 5.00	6.96 + 4.00	3.45 + 4.00
TKW /g	13.78 + 0.31	13.68 + 0.60	13.78 + 0.79	15.92 + 0.41	15.10 + 0.54	14.83 + 0.48	16.86 + 0.58	16.75 + 0.54	15.92 + 0.57

Notes: Numbers denote the average of empty seed rate, shrunken seeds rate and TKW in each sampling plots.

$$\text{Empty seed rate}(\%) = \frac{\text{Theorical seed number of single infructescence} - \text{Practical seed number of single infructescence}}{\text{Theorical seed number of single infructescence}} \times 100\%$$

$$\text{Shrunken seeds rate}(\%) = \frac{\text{Shrunken seeds number of single infructescence}}{\text{Seeds number of single infructescence}} \times 100\%$$
